# 7-(4-Methoxy­phen­yl)-5-methyl-9-phenyl-7*H*-pyrrolo[2′,3′:4,5]pyrimido[1,6-*d*]tetrazole

**DOI:** 10.1107/S1600536809053653

**Published:** 2009-12-19

**Authors:** Mukesh M. Jotani, Rina D. Shah, Jerry P. Jasinski

**Affiliations:** a Bhavan’s Sheth R.A. College of Science, Ahmedabd, Gujarat 380 001, India; b M. G. Science Institute, Navrangpura, Ahmedabad, Gujarat 380 009, India; c Department of Chemistry, Keene State College, 229 Main Street, Keene, NH 03435-2001, USA

## Abstract

The title compound, C_20_H_16_N_6_O, is composed of a tetra­zolo ring and a 4-methoxy­phenyl and a benzene-substituted pyrrole ring at the 7 and 9 positions fused to a pyrimidine ring in a nearly planar fashion [maximum deviation of 0.018 (1) Å for the fused ring system]. A methyl group at the 5 position is also in the plane of the hetero cyclic system. The dihedral angle between the mean planes of the benzene and 4-methoxy­phenyl rings is 40.4 (2)°. The dihedral angles between the mean planes of the pyrimidine and the benzene and 4-methoxy­phenyl rings are 15.6 (5)° and 52.6 (7)°, respectively. A weak intra­molecular C—H⋯N hydrogen bond inter­action, which forms an *S*(7) graph-set motif, helps to establish the relative conformations of the tetrazolo and benzene rings. In the crystal, weak inter­molecular C—H⋯O, C—H⋯π and π–π stacking inter­actions [centroid–centroid distances = 3.5270 (16), 3.5113 (16), 3.7275 (17) and 3.7866 (17) Å] link the mol­ecules into a two-dimensional array obliquely parallel to (101) and propagating along the *b* axis.

## Related literature

For the biological activity of fused tetra­zolopyrimidines, see: Wilkinson (1992 ▶); Omer *et al.* (1991 ▶); Schram *et al.* (1975 ▶). Fused pyrimidines with a halogen at the 2- or 4- position seem to be more labile towards a nucleophilic substitution reaction with reagents such as piperadine, piperazine, morpholine, hydrazine and azides, forming potent bi- and triheterocycles, see: Dave & Shah (2000 ▶, 2002 ▶); Peinador *et al.* (1992 ▶); Schneller & Clough (1992 ▶); Shishoo & Jain (1992 ▶). For the importance of the reduction of tetra­zolopyrimidines *via* azido­lysis in the development of synthetically important 4-amino­pyrimidines, see: Shishoo & Jain (1992 ▶); Hand & Backer (1984 ▶). For nucleophilic substitution reactions in pyrrolo[2,3-*e*] pyrimidines, see: Dave & Shah (2002 ▶); Ali & Swealan (1992 ▶). For related structures, see: Jotani & Baldaniya (2007 ▶, 2008 ▶); Hou *et al.* (2009 ▶); Baldaniya & Jotani (2008 ▶); Malone *et al.* (1997 ▶). For the synthesis, see: Shah (2009 ▶). For hydrogen-bond motifs, see: Bernstein *et al.* (1995 ▶). For tetrazolo ring formation, see: Bourgurgnon *et al.* (1975 ▶); Robba *et al.* (1975 ▶).
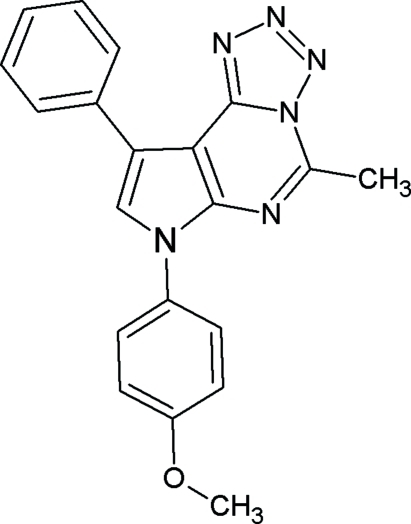


## Experimental

### 

#### Crystal data

C_20_H_16_N_6_O
*M* *_r_* = 356.39
Monoclinic, 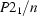

*a* = 13.738 (2) Å
*b* = 7.032 (3) Å
*c* = 19.4350 (3) Å
β = 110.217 (2)°
*V* = 1761.9 (8) Å^3^
*Z* = 4
Mo *K*α radiation
μ = 0.09 mm^−1^
*T* = 293 K
0.47 × 0.35 × 0.2 mm

#### Data collection

Bruker Kappa APEXII CCD diffractometer
Absorption correction: multi-scan (*SADABS*; Sheldrick, 2004 ▶) *T*_min_ = 0.96, *T*_max_ = 0.98
42323 measured reflections
5137 independent reflections
3694 reflections with *I* > 2σ(*I*)
*R*_int_ = 0.030

#### Refinement

*R*[*F* ^2^ > 2σ(*F* ^2^)] = 0.045
*wR*(*F* ^2^) = 0.129
*S* = 1.04
5137 reflections
247 parameters
H-atom parameters constrained
Δρ_max_ = 0.29 e Å^−3^
Δρ_min_ = −0.20 e Å^−3^

### 

Data collection: *APEX2* (Bruker, 2004 ▶); cell refinement: *APEX2* and *SAINT* (Bruker, 2004 ▶); data reduction: *SAINT* and *XPREP* (Bruker, 2004 ▶); program(s) used to solve structure: *SIR2004* (Burla *et al.*, 2003 ▶); program(s) used to refine structure: *SHELXL97* (Sheldrick, 2008 ▶); molecular graphics: *ORTEP-32* and *PLATON* (Spek, 2009 ▶); software used to prepare material for publication: *SHELXL97*.

## Supplementary Material

Crystal structure: contains datablocks global, I. DOI: 10.1107/S1600536809053653/gw2072sup1.cif

Structure factors: contains datablocks I. DOI: 10.1107/S1600536809053653/gw2072Isup2.hkl

Additional supplementary materials:  crystallographic information; 3D view; checkCIF report

## Figures and Tables

**Table 1:** Hydrogen-bond geometry (Å, °)

*D*—H⋯*A*	*D*—H	H⋯*A*	*D*⋯*A*	*D*—H⋯*A*
C13—H13⋯N6	0.95	2.38	3.2159 (18)	146
C12—H12⋯O1^i^	0.95	2.57	3.3902 (17)	144
C7—H7*B*⋯*Cg*4^ii^	0.96	2.90	3.620 (2)	132
C7—H7*C*⋯*Cg*4^iii^	0.96	2.69	3.554 (2)	150

Symmetry codes: (i) 
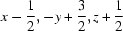
; (ii) 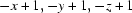; (iii) 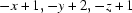. *Cg*4 is the centroid of the C8–C13. ring.

**Fig. 1.**
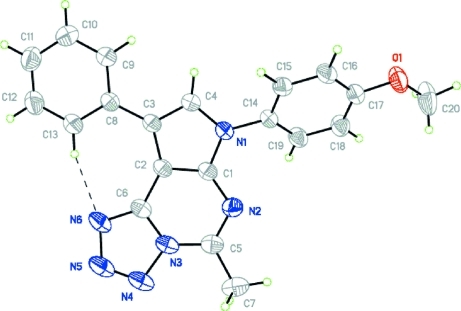
The molecular structure of (I), showing the atom labelling scheme and 50% probability displacement ellipsoids. The dashed line represents a weak intramolecular C–H···N hydrogen bond interaction.

**Fig. 2.**
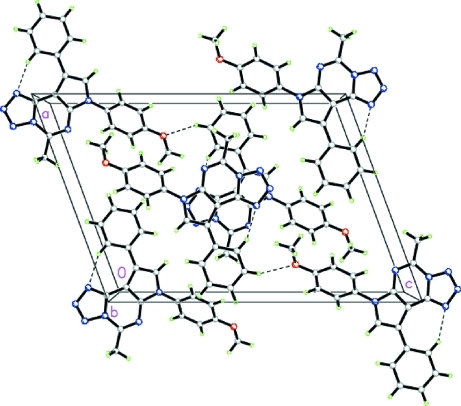
Crystal packing of (I), showing weak intramolecular C–H···N and intermolecular C–H···O hydrogen bond interactions as dashed lines.

**Fig. 3.**
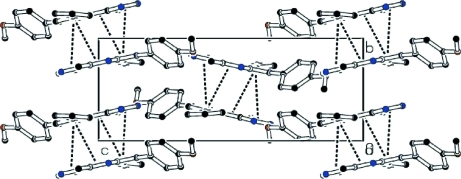
The molecular packing of (I), showing π–π stacking interactions as dashed lines forming chains of molecules along [1 0 1] plane if the unit cell.

## supplementary crystallographic information

### Comment

Fused tetrazolopyrimidines are important to the activities of a variety of biological substances (Willkinson, 1992; Omer *et al.*, 1991; Schram *et al.*, 1975). Moreover, fused pyrimidines having a halogen at the 2- or 4- position seem to be more labile towards a nucleophilic substitution reaction with reagents such as piperadine, piperazine, morpholine, hydrazine and azides to form potent bi and triheterocycles (Dave & Shah, 2002; Peinador *et al.*, 1992; Schneller & Clough, 1992; Shishoo & Jain, 1992). The reduction of tetrazolopyrimidines *via* azidolysis studies have been shown to be to attractive to development of synthetically important 4-aminopyrimidines (Shishoo & Jain, 1992; Hand & Backer, 1984). The treatment of sodium azide with 4-chloropyrrolo[2,3-*e*] pyrimidine can result the formation of either an azido group or tetrazole ring upon a fused pyrimidine ring. Such nucleophilic substitution reactions have rarely been attempted in pyrrolo[2,3-e] pyrimidines (Dave & Shah, 2002; Ali & Swealan, 1992). In view of the importance of these molecules, a crystal structure of the title compound, C_20_H_16_N_6_O, (I) has been determined.

The title compound, C_20_H_16_N_6_O, (I), is composed of a tetrazolo ring and a 4-methoxyphenyl and benzene substituted pyrrole ring at the 7 and 9 position fused to a pyrimidine ring in a nearly planar fashion (Fig. 1). The r.m.s.deviation of atoms of the fused ring from the mean plane through the heterotricyclic system is 0.0085 Å, with a maximum deviation of -0.018 (1) and 0.013 (1) Å for atoms C2 and C3 respectively. Bond lengths and angles for the fused pyrrole and tetrazole rings in (I) are normal and similar to that observed for a related structure. The dihedral angles between the mean planes of fused pyrimidine and tetrazole rings with that of the pyrrole ring are 1.26 (6) ° and 1.13 (7)°, respectively. A methyl group at the 5 position is also in the plane of the pyrimidine ring. The dihedral angle between the mean planes of the benzene and 4-methoxyphenyl rings is 40.4 (2)°. The angles between the mean planes of the pyrimidine and the benzene and 4-methoxyphenyl rings are 15.6 (5)° and 52.6 (7)°, respectively. A weak intramolecular C13–H13···N6 hydrogen bond interaction, which forms an S(7) graph set, helps stabilize the separation angle between the tetrazolo and benzene rings. Weak intermolecular C12–H12···O1 (Fig. 2), C–H···π-ring (C7–H7B(H7C)···*Cg*4 [= 3.620 (2) (3.554 (2) Å; 1 - *x*, 1 - *y*, 1 - *z* (1 - *x*, 2 - *y*, 1 - *z*); where *Cg*4 = C8–C13 ring centroid; Table 1] and π–π [*Cg*1···*Cg*2; = 3.5270 (16)Å & 3.5113 (16) Å,1 - *x*, 1 - *y*, 1 - *x* & 1 - *x*, 2 - *y*, 1 - *z*; *Cg*2···*Cg*3; = 3.7275 (17) Å, 1 - *x*, 2 - *y*, 1 - *z*; *Cg*3···*Cg*1; = 3.7866 (17) Å, 1 - *x*, 1 - *y*, 1 - *z*; where *Cg*1 = N1/C1–C4, *Cg*2 = N3–N6/C6, *Cg*3 = N2/C1–C5] stacking interactions (Fig. 3) help to link the molecules into a 2-D array  obliquely parallel to (101) and propagating along the b axis.

### Experimental

The title compound was synthesized according to method of Shah (2009). A mixture of sodium azide (0.011 mole), ammonium chloride (0.011 mole) and 2-methyl-5-phenyl-7-(4-methoxyphenyl)-4-chloro-7*H*-pyrrolo[2,3-*d*]pyrimidine (0.01 mole) in DMSO (20 ml) was stirred for for 2 h at 363 K to obtain the title compound (I). Colorless platlike single crystals, suitable for X-ray diffraction were grown from a solution of 1,4-dioxane.

### Refinement

All of the H atoms were placed in their calculated positions and then refined using the riding model with C—H = 0.95–0.98 Å, and with *U*_iso_(H) = 1.18–1.50*U*_eq_(C).

### Crystal data

**Table:**

C_20_H_16_N_6_O	*F*(000) = 744
*M**_r_* = 356.39	*D*_x_ = 1.344 Mg m^−^^3^
Monoclinic, *P*2_1_/*n*	Mo *K*α radiation, λ = 0.71073 Å
Hall symbol: -P 2yn	Cell parameters from 5176 reflections
*a* = 13.738 (2) Å	θ = 2.2–31.3°
*b* = 7.032 (3) Å	µ = 0.09 mm^−^^1^
*c* = 19.4350 (3) Å	*T* = 293 K
β = 110.217 (2)°	Plate, yellow
*V* = 1761.9 (8) Å^3^	0.47 × 0.35 × 0.2 mm
*Z* = 4	

### Data collection

**Table:**

Bruker Kappa APEXII CCD diffractometer	5137 independent reflections
Radiation source: fine-focus sealed tube	3694 reflections with *I* > 2σ(*I*)
graphite	*R*_int_ = 0.030
ω and φ scan	θ_max_ = 30.0°, θ_min_ = 1.6°
Absorption correction: multi-scan (*SADABS*; Sheldrick, 2004)	*h* = −19→19
*T*_min_ = 0.96, *T*_max_ = 0.98	*k* = −4→9
42323 measured reflections	*l* = −27→27

### Refinement

**Table:**

Refinement on *F*^2^	Secondary atom site location: difference Fourier map
Least-squares matrix: full	Hydrogen site location: inferred from neighbouring sites
*R*[*F*^2^ > 2σ(*F*^2^)] = 0.045	H-atom parameters constrained
*wR*(*F*^2^) = 0.129	*w* = 1/[σ^2^(*F*_o_^2^) + (0.0656*P*)^2^ + 0.2469*P*] where *P* = (*F*_o_^2^ + 2*F*_c_^2^)/3
*S* = 1.03	(Δ/σ)_max_ = 0.001
5137 reflections	Δρ_max_ = 0.29 e Å^−^^3^
247 parameters	Δρ_min_ = −0.20 e Å^−^^3^
0 restraints	Extinction correction: *SHELXL97* (Sheldrick, 2008), Fc^*^=kFc[1+0.001xFc^2^λ^3^/sin(2θ)]^-1/4^
Primary atom site location: structure-invariant direct methods	Extinction coefficient: 0.0104 (12)

### Special details

**Table:**

Geometry. All e.s.d.'s (except the e.s.d. in the dihedral angle between two l.s. planes) are estimated using the full covariance matrix. The cell e.s.d.'s are taken into account individually in the estimation of e.s.d.'s in distances, angles and torsion angles; correlations between e.s.d.'s in cell parameters are only used when they are defined by crystal symmetry. An approximate (isotropic) treatment of cell e.s.d.'s is used for estimating e.s.d.'s involving l.s. planes.
Refinement. Refinement of *F*^2^ against ALL reflections. The weighted *R*-factor *wR* and goodness of fit *S* are based on *F*^2^, conventional *R*-factors *R* are based on *F*, with *F* set to zero for negative *F*^2^. The threshold expression of *F*^2^ > σ(*F*^2^) is used only for calculating *R*-factors(gt) *etc*. and is not relevant to the choice of reflections for refinement. *R*-factors based on *F*^2^ are statistically about twice as large as those based on *F*, and *R*- factors based on ALL data will be even larger.

### Fractional atomic coordinates and isotropic or equivalent isotropic displacement parameters (Å^2^)

**Table:**

	*x*	*y*	*z*	*U*_iso_*/*U*_eq_	
N1	0.49323 (7)	0.66669 (14)	0.35550 (5)	0.0364 (2)	
N2	0.64444 (8)	0.71967 (13)	0.46114 (6)	0.0390 (2)	
N3	0.60830 (8)	0.78499 (13)	0.56626 (5)	0.0387 (2)	
N4	0.63253 (10)	0.82763 (16)	0.63905 (6)	0.0503 (3)	
N5	0.54477 (10)	0.84057 (18)	0.64892 (6)	0.0553 (3)	
N6	0.46241 (9)	0.80796 (15)	0.58676 (5)	0.0461 (3)	
O1	0.67511 (8)	0.63011 (14)	0.14173 (5)	0.0557 (3)	
C1	0.54006 (9)	0.70521 (14)	0.42843 (6)	0.0342 (2)	
C2	0.46308 (9)	0.72839 (14)	0.45931 (6)	0.0322 (2)	
C3	0.36448 (9)	0.70460 (14)	0.40206 (6)	0.0331 (2)	
C4	0.38831 (9)	0.66682 (16)	0.34039 (6)	0.0372 (2)	
H4	0.3383	0.6437	0.2933	0.045*	
C5	0.67850 (10)	0.75945 (15)	0.53047 (7)	0.0400 (3)	
C6	0.50261 (9)	0.77276 (14)	0.53513 (6)	0.0350 (2)	
C7	0.79065 (11)	0.7757 (2)	0.57366 (8)	0.0538 (3)	
H7A	0.8307	0.7651	0.5408	0.081*	
H7B	0.8109	0.6735	0.6102	0.081*	
H7C	0.8044	0.8991	0.5986	0.081*	
C8	0.25890 (9)	0.71760 (15)	0.40362 (6)	0.0348 (2)	
C9	0.17475 (10)	0.73306 (18)	0.33836 (7)	0.0437 (3)	
H9	0.1867	0.7361	0.2931	0.052*	
C10	0.07452 (11)	0.7441 (2)	0.33822 (8)	0.0531 (3)	
H10	0.0183	0.7540	0.2931	0.064*	
C11	0.05569 (11)	0.7407 (2)	0.40303 (9)	0.0572 (4)	
H11	−0.0134	0.7493	0.4030	0.069*	
C12	0.13717 (12)	0.7249 (2)	0.46801 (9)	0.0559 (4)	
H12	0.1242	0.7217	0.5129	0.067*	
C13	0.23768 (11)	0.71372 (18)	0.46865 (7)	0.0444 (3)	
H13	0.2932	0.7032	0.5141	0.053*	
C14	0.54240 (9)	0.65119 (16)	0.30183 (6)	0.0352 (2)	
C15	0.50818 (9)	0.76648 (17)	0.24049 (6)	0.0409 (3)	
H15	0.4533	0.8542	0.2348	0.049*	
C16	0.55355 (10)	0.75407 (18)	0.18782 (7)	0.0437 (3)	
H16	0.5294	0.8320	0.1454	0.052*	
C17	0.63448 (9)	0.62818 (17)	0.19641 (6)	0.0397 (3)	
C18	0.66942 (9)	0.51457 (18)	0.25825 (7)	0.0430 (3)	
H18	0.7254	0.4291	0.2646	0.052*	
C19	0.62275 (9)	0.52564 (17)	0.31077 (6)	0.0413 (3)	
H19	0.6461	0.4467	0.3530	0.050*	
C20	0.75810 (14)	0.5043 (3)	0.14708 (10)	0.0782 (6)	
H20A	0.8168	0.5324	0.1918	0.117*	
H20B	0.7795	0.5207	0.1043	0.117*	
H20C	0.7354	0.3728	0.1488	0.117*	

### Atomic displacement parameters (Å^2^)

**Table:**

	*U*^11^	*U*^22^	*U*^33^	*U*^12^	*U*^13^	*U*^23^
N1	0.0378 (5)	0.0443 (5)	0.0276 (5)	0.0018 (4)	0.0121 (4)	−0.0010 (4)
N2	0.0382 (5)	0.0396 (5)	0.0362 (5)	0.0018 (4)	0.0092 (4)	0.0011 (4)
N3	0.0492 (6)	0.0345 (4)	0.0271 (5)	−0.0023 (4)	0.0064 (4)	0.0001 (3)
N4	0.0663 (8)	0.0509 (6)	0.0272 (5)	−0.0076 (5)	0.0080 (5)	−0.0035 (4)
N5	0.0717 (8)	0.0613 (7)	0.0300 (6)	−0.0110 (6)	0.0139 (5)	−0.0068 (5)
N6	0.0620 (7)	0.0495 (6)	0.0284 (5)	−0.0074 (5)	0.0175 (5)	−0.0042 (4)
O1	0.0641 (6)	0.0690 (6)	0.0459 (5)	0.0180 (5)	0.0340 (5)	0.0111 (4)
C1	0.0401 (6)	0.0329 (5)	0.0285 (5)	0.0015 (4)	0.0105 (4)	0.0016 (4)
C2	0.0406 (6)	0.0288 (4)	0.0272 (5)	0.0002 (4)	0.0118 (4)	0.0018 (4)
C3	0.0388 (6)	0.0327 (5)	0.0278 (5)	0.0006 (4)	0.0115 (4)	0.0016 (4)
C4	0.0365 (6)	0.0436 (6)	0.0298 (5)	0.0000 (4)	0.0095 (5)	−0.0017 (4)
C5	0.0437 (7)	0.0334 (5)	0.0373 (6)	0.0010 (4)	0.0069 (5)	0.0014 (4)
C6	0.0458 (6)	0.0289 (5)	0.0293 (5)	−0.0020 (4)	0.0115 (5)	0.0017 (4)
C7	0.0451 (7)	0.0510 (7)	0.0520 (8)	−0.0023 (5)	−0.0001 (6)	−0.0040 (6)
C8	0.0407 (6)	0.0320 (5)	0.0326 (6)	−0.0005 (4)	0.0137 (5)	−0.0007 (4)
C9	0.0426 (7)	0.0532 (7)	0.0346 (6)	0.0003 (5)	0.0124 (5)	−0.0012 (5)
C10	0.0404 (7)	0.0647 (8)	0.0496 (8)	0.0015 (6)	0.0096 (6)	−0.0037 (6)
C11	0.0430 (8)	0.0684 (9)	0.0646 (10)	−0.0028 (6)	0.0244 (7)	−0.0089 (7)
C12	0.0543 (8)	0.0711 (9)	0.0512 (8)	−0.0043 (7)	0.0297 (7)	−0.0068 (6)
C13	0.0464 (7)	0.0536 (7)	0.0349 (6)	−0.0008 (5)	0.0161 (5)	−0.0016 (5)
C14	0.0369 (6)	0.0420 (5)	0.0277 (5)	−0.0006 (4)	0.0124 (4)	−0.0023 (4)
C15	0.0381 (6)	0.0512 (6)	0.0327 (6)	0.0103 (5)	0.0113 (5)	0.0035 (5)
C16	0.0448 (7)	0.0550 (7)	0.0312 (6)	0.0087 (5)	0.0129 (5)	0.0090 (5)
C17	0.0416 (6)	0.0474 (6)	0.0337 (6)	0.0020 (5)	0.0174 (5)	0.0000 (4)
C18	0.0451 (7)	0.0448 (6)	0.0426 (7)	0.0110 (5)	0.0195 (5)	0.0049 (5)
C19	0.0472 (7)	0.0433 (6)	0.0348 (6)	0.0075 (5)	0.0158 (5)	0.0069 (4)
C20	0.0943 (13)	0.0825 (11)	0.0864 (12)	0.0367 (10)	0.0674 (11)	0.0245 (9)

### Geometric parameters (Å, °)

**Table:**

N1—C1	1.3661 (14)	C8—C9	1.3942 (17)
N1—C4	1.3680 (15)	C9—C10	1.3783 (18)
N1—C14	1.4294 (14)	C9—H9	0.9500
N2—C5	1.2951 (16)	C10—C11	1.371 (2)
N2—C1	1.3567 (15)	C10—H10	0.9500
N3—C6	1.3691 (16)	C11—C12	1.372 (2)
N3—N4	1.3699 (14)	C11—H11	0.9500
N3—C5	1.3819 (17)	C12—C13	1.379 (2)
N4—N5	1.2879 (17)	C12—H12	0.9500
N5—N6	1.3591 (16)	C13—H13	0.9500
N6—C6	1.3254 (15)	C14—C19	1.3768 (16)
O1—C17	1.3606 (13)	C14—C15	1.3828 (16)
O1—C20	1.4183 (17)	C15—C16	1.3730 (17)
C1—C2	1.3942 (16)	C15—H15	0.9500
C2—C6	1.4178 (15)	C16—C17	1.3854 (17)
C2—C3	1.4351 (15)	C16—H16	0.9500
C3—C4	1.3730 (15)	C17—C18	1.3835 (16)
C3—C8	1.4640 (16)	C18—C19	1.3825 (16)
C4—H4	0.9500	C18—H18	0.9500
C5—C7	1.4824 (18)	C19—H19	0.9500
C7—H7A	0.9800	C20—H20A	0.9800
C7—H7B	0.9800	C20—H20B	0.9800
C7—H7C	0.9800	C20—H20C	0.9800
C8—C13	1.3920 (17)		
			
C1—N1—C4	107.82 (9)	C10—C9—H9	119.3
C1—N1—C14	126.90 (10)	C8—C9—H9	119.3
C4—N1—C14	124.89 (9)	C11—C10—C9	120.20 (14)
C5—N2—C1	116.55 (11)	C11—C10—H10	119.9
C6—N3—N4	108.19 (10)	C9—C10—H10	119.9
C6—N3—C5	125.97 (10)	C10—C11—C12	119.60 (13)
N4—N3—C5	125.83 (11)	C10—C11—H11	120.2
N5—N4—N3	105.27 (10)	C12—C11—H11	120.2
N4—N5—N6	112.98 (11)	C11—C12—C13	120.58 (13)
C6—N6—N5	105.53 (11)	C11—C12—H12	119.7
C17—O1—C20	118.22 (10)	C13—C12—H12	119.7
N2—C1—N1	122.92 (10)	C12—C13—C8	120.93 (13)
N2—C1—C2	128.74 (10)	C12—C13—H13	119.5
N1—C1—C2	108.33 (10)	C8—C13—H13	119.5
C1—C2—C6	113.45 (10)	C19—C14—C15	120.24 (10)
C1—C2—C3	107.80 (9)	C19—C14—N1	121.15 (10)
C6—C2—C3	138.71 (11)	C15—C14—N1	118.61 (10)
C4—C3—C2	104.68 (10)	C16—C15—C14	119.92 (11)
C4—C3—C8	124.54 (10)	C16—C15—H15	120.0
C2—C3—C8	130.77 (10)	C14—C15—H15	120.0
N1—C4—C3	111.35 (10)	C15—C16—C17	120.19 (11)
N1—C4—H4	124.3	C15—C16—H16	119.9
C3—C4—H4	124.3	C17—C16—H16	119.9
N2—C5—N3	119.19 (11)	O1—C17—C18	124.85 (11)
N2—C5—C7	122.47 (12)	O1—C17—C16	115.35 (10)
N3—C5—C7	118.33 (11)	C18—C17—C16	119.78 (11)
N6—C6—N3	108.03 (10)	C19—C18—C17	119.91 (11)
N6—C6—C2	135.87 (11)	C19—C18—H18	120.0
N3—C6—C2	116.10 (10)	C17—C18—H18	120.0
C5—C7—H7A	109.5	C14—C19—C18	119.94 (11)
C5—C7—H7B	109.5	C14—C19—H19	120.0
H7A—C7—H7B	109.5	C18—C19—H19	120.0
C5—C7—H7C	109.5	O1—C20—H20A	109.5
H7A—C7—H7C	109.5	O1—C20—H20B	109.5
H7B—C7—H7C	109.5	H20A—C20—H20B	109.5
C13—C8—C9	117.35 (11)	O1—C20—H20C	109.5
C13—C8—C3	122.51 (11)	H20A—C20—H20C	109.5
C9—C8—C3	120.13 (10)	H20B—C20—H20C	109.5
C10—C9—C8	121.33 (12)		
			
C6—N3—N4—N5	−0.45 (12)	C5—N3—C6—C2	0.47 (15)
C5—N3—N4—N5	179.34 (10)	C1—C2—C6—N6	179.44 (12)
N3—N4—N5—N6	0.33 (14)	C3—C2—C6—N6	1.9 (2)
N4—N5—N6—C6	−0.08 (14)	C1—C2—C6—N3	−0.34 (13)
C5—N2—C1—N1	179.05 (10)	C3—C2—C6—N3	−177.88 (11)
C5—N2—C1—C2	0.35 (17)	C4—C3—C8—C13	165.29 (11)
C4—N1—C1—N2	−178.52 (10)	C2—C3—C8—C13	−15.64 (17)
C14—N1—C1—N2	−5.42 (17)	C4—C3—C8—C9	−14.18 (16)
C4—N1—C1—C2	0.41 (12)	C2—C3—C8—C9	164.89 (11)
C14—N1—C1—C2	173.51 (10)	C13—C8—C9—C10	0.00 (17)
N2—C1—C2—C6	−0.04 (16)	C3—C8—C9—C10	179.50 (11)
N1—C1—C2—C6	−178.89 (9)	C8—C9—C10—C11	0.3 (2)
N2—C1—C2—C3	178.25 (10)	C9—C10—C11—C12	−0.5 (2)
N1—C1—C2—C3	−0.60 (11)	C10—C11—C12—C13	0.4 (2)
C1—C2—C3—C4	0.54 (11)	C11—C12—C13—C8	−0.2 (2)
C6—C2—C3—C4	178.17 (12)	C9—C8—C13—C12	−0.05 (17)
C1—C2—C3—C8	−178.67 (10)	C3—C8—C13—C12	−179.54 (11)
C6—C2—C3—C8	−1.0 (2)	C1—N1—C14—C19	55.88 (16)
C1—N1—C4—C3	−0.06 (13)	C4—N1—C14—C19	−132.14 (12)
C14—N1—C4—C3	−173.33 (10)	C1—N1—C14—C15	−123.61 (12)
C2—C3—C4—N1	−0.30 (12)	C4—N1—C14—C15	48.36 (16)
C8—C3—C4—N1	178.97 (9)	C19—C14—C15—C16	0.85 (18)
C1—N2—C5—N3	−0.24 (15)	N1—C14—C15—C16	−179.66 (11)
C1—N2—C5—C7	178.70 (10)	C14—C15—C16—C17	−0.88 (19)
C6—N3—C5—N2	−0.17 (16)	C20—O1—C17—C18	1.4 (2)
N4—N3—C5—N2	−179.92 (10)	C20—O1—C17—C16	−179.88 (14)
C6—N3—C5—C7	−179.15 (10)	C15—C16—C17—O1	−178.74 (12)
N4—N3—C5—C7	1.10 (16)	C15—C16—C17—C18	0.1 (2)
N5—N6—C6—N3	−0.22 (12)	O1—C17—C18—C19	179.42 (12)
N5—N6—C6—C2	179.99 (12)	C16—C17—C18—C19	0.70 (19)
N4—N3—C6—N6	0.42 (12)	C15—C14—C19—C18	−0.04 (18)
C5—N3—C6—N6	−179.37 (10)	N1—C14—C19—C18	−179.52 (11)
N4—N3—C6—C2	−179.74 (9)	C17—C18—C19—C14	−0.73 (19)

### Hydrogen-bond geometry (Å, °)

**Table:**

*D*—H···*A*	*D*—H	H···*A*	*D*···*A*	*D*—H···*A*
C13—H13···N6	0.95	2.38	3.2159 (18)	146
C12—H12···O1^i^	0.95	2.57	3.3902 (17)	144
C7—H7B···Cg4^ii^	0.96	2.90	3.620 (2)	132
C7—H7C···Cg4^iii^	0.96	2.69	3.554 (2)	150

Symmetry codes: (i) *x*−1/2, −*y*+3/2, *z*+1/2; (ii) −*x*+1, −*y*+1, −*z*+1; (iii) −*x*+1, −*y*+2, −*z*+1.
